# Taxonomic Revision of the South American Genus *Eudius* and First Insights into the Phylogeny of the Tribe Eudiagogini (Curculionidae: Entiminae)

**DOI:** 10.3390/insects16121278

**Published:** 2025-12-16

**Authors:** Judy A. Rincón, María Guadalupe del Río, Adriana E. Marvaldi

**Affiliations:** 1 División Entomología, Facultad de Ciencias Naturales y Museo, Universidad Nacional de La Plata, CONICET (National Scientific and Technical Research Council), Paseo del Bosque s/n, La Plata B1900FWA, Argentina; j.andrearincon@gmail.com; 2 Grupo Sistemática de Insectos Agronomía SIA, Museo Entomológico UNAB, Facultad de Ciencias Agrarias, Universidad Nacional de Colombia, Cra. 30 #45-03, Bogotá 111321, Colombia

**Keywords:** broad-nosed weevils, *Chileudius*, genetic data, morphology, neotropics, taxonomy

## Abstract

The genus *Eudius* Schoenherr is classified in the broad-nosed weevil tribe Eudiagogini (Entiminae) and harbors two species, *Eudius quadrisignatus* Gyllenhal and *Eudius jocosus* Fahraeus, which are only known from their original descriptions. It is endemic to the Brazilian Atlantic Forest, which is one of the most threatened biomes in the world despite being a biodiversity hotspot. In this contribution, and as part of a wider systematic and phylogenetic study on tribe Eudiagogini, we performed a taxonomic revision of the genus *Eudius* and made preliminary phylogenetic analyses of Eudiagogini based on morphology and molecular evidence. Specimens from seven collections in Argentina, Brazil, and Europe were examined. Diagnosis and redescription of the genus and its species are provided, along with photographs of habits, and illustrations of diagnostic characters and new geographic distribution data. Additionally, a lectotype is designated for each species. The morphology-based phylogenetic analysis was performed under maximum parsimony, using 60 characters from adults coded for representative species from eight genera of Eudiagogini and other related tribes of Entiminae. As a result, monophyly of the genus *Eudius* and its placement within the tribe Eudiagogini are confirmed, while placement of the genus *Chileudius* Kuschel in Eudiagogini is refuted. A first molecular phylogenetic analysis of the tribe was also designed, using DNA sequences (of the COI barcode and two ribosomal markers) available for some representatives of Eudiagogini and outgroup taxa, analyzed under parsimony and maximum likelihood. The molecular results are consistent with morphology in recovering a monophyletic tribe Eudiagogini, excluding the genus *Chileudius*, which is now placed as *incertae sedis* in Entiminae, pending further analyses. Informative characters within the tribe are discussed, with *Eudius* supported as a clade by the basally connate tarsal claws and by the sclerites present in the bursa of female genitalia. Synapomorphies justifying the revised concept of Eudiagogini as a natural tribe are highlighted, like the presence of a cavernous prementum and the metaventrite with a spine-like swelling anterior to each metacoxa.

## Simple Summary

Broad-nosed weevils of the genus *Eudius* Schoenherr (Curculionidae, Eudiagogini) are endemic to the Brazilian Atlantic Forest, a biome of critical conservation concern. Knowledge of its two species is scarce beyond their original descriptions. In this study, we performed a taxonomic review providing revised descriptions and diagnoses of the genus and its species, along with updated distribution data. In addition, we undertook a preliminary phylogenetic analysis of the tribe Eudiagogini, based on adult morphological characters and available genetic data. The morphology-based phylogenetic analysis corroborates the monophyly of the genus *Eudius*, provides support for its placement within Eudiagogini, and clarifies generic relationships. The molecular-based analyses using available DNA sequences (of COI barcode and two ribosomal markers for a subset of the taxa) provide results consistent with morphology, worth mentioning the monophyletic concept of the tribe Eudiagogini, excluding the genus *Chileudius* Kuschel. We expect that our study will contribute to a better understanding of the uniquely diverse Neotropical weevils and help advance towards a natural tribal classification of the hyperdiverse subfamily Entiminae.

## 1. Introduction

The broad-nosed weevil tribe Eudiagogini LeConte 1874 (Curculionidae, Entiminae) contains 98 extant species in 10 genera, distributed in the American Continent, with the highest diversity in the Neotropics [1,2,3]. The Eudiagogini (=Promecopini) have been traditionally placed in the “Leptopiinae” in old classifications (e.g., [1,2,4,5]), together with the Cylydrorhinini, Entimini, Lordopini (=Hypsonotini), Ophryastini, Premnotrypini, and the Leptopiini. The latter name has been synonymized, with Strangaliodini and Pantopoeini being the tribal names currently used, respectively, for the South American and Indo-Australian genera formerly in Leptopiini [6]; see [7] for more details (p. 452). These weevils are joined mainly by the presence of postocular lobes and lateral antennal scrobes.

The recognition of monophyletic tribes and genera towards a natural classification of the hyperdiverse subfamily Entiminae remains among the major challenges of weevil systematics [6,7,8,9]. Eudiagogini is one of the less studied tribes; only its type genus has been taxonomically revised [10,11]. The other nine genera classified in Eudiagogini [3] remain scarcely known beyond the original descriptions. Their monophyly and tribal placement have never been formally tested, and, for example, the assignment to Eudiagogini of the genus *Chileudius* Kuschel is particularly doubtful [12]. There are no phylogenetic studies on Eudiagogini and the tribe is not sampled (e.g., [6,13]) and is poorly represented in phylogenetic studies of weevils (e.g., [9]).

Among the genera of Eudiagogini, *Eudius* Schoenherr 1834 is of particular interest due to being endemic to one of the most threatened biomes of the world, the Brazilian Atlantic Forest. It comprises two species, *Eudius quadrisignatus* Gyllenhal 1834 and *Eudius jocosus* Fahraeus 1840, grouped mainly by the presence of small, basally connate tarsal claws. Its species are also characterized by a broadly oval body, prothorax with postocular lobes, pronotum with three light-colored bands alternating with two wider dark bands, and elytra with a V-shaped color pattern continuing with discontinuous longitudinal bands. The genus lacks a formal review, and there is little information on its species. Considering the relevance of its distribution in a biome of major conservation concern and recognized as a biodiversity hotspot [14], this work provides a taxonomic review of the genus *Eudius* with new data on its geographic distribution and evaluates its monophyly and phylogenetic position in the tribe Eudiagogini, through a cladistic analysis using adult morphological characters. Moreover, this study uses additional evidence from DNA sequence data towards a monophyletic concept of the tribe Eudiagogini.

## 2. Materials and Methods

### 2.1. Specimen Repositories

For the taxonomic study of *Eudius*, seventy-six specimens were examined, including the material type of both species, from seven collections in Argentina, Brazil, and Europe.

MACN: Museo Argentino de Ciencias Naturales Bernardino Rivadavia, Buenos Aires, Argentina
MZSP: Museu de Zoologia Universidade de São Paulo, Brazil
MNHN: Muséum National d’Histoire Naturelle, Paris, France
NHMUK: Natural History Museum, London, United Kingdom
MFN: Museum für Naturkunde, Berlin, Germany
MTD: Senckenberg Naturhistorische Sammlungen (Staatliches Museum für Tierkunde), Dresden, Germany
NHRS: Naturhistoriska Riksmuseet, Stockholm, Sweden

### 2.2. Morphological Study

External and internal structures were observed and measured with a Nikon SMZ800 (Tokyo, Japan) stereomicroscope. Photographs were taken with a Leica MZ16F (Wetzlar, Germany) stereomicroscope and a JVC KY-F75U (Hachioji, Japan) camera; line drawings were performed by tracing over photographs using a drawing software Inkscape v1.4.2.

Dissections were performed as follows: dry specimens were softened in a wet chamber before dissection, terminalia with genital structures were placed in a solution of KOH 10% until cleared (usually 24 h at room temperature), then immersed in a 5% aqueous solution of acetic acid (distilled vinegar) to neutralize potash, then rinsed in distilled water and finally placed in glycerin.

Morphological terminology follows the study of Oberprieler & Zimmerman [15], and the glossary of weevil characters [16].

Measurements, with their abbreviations, are as follows:

TL (Total length): measured in dorsal view from anterior margin of pronotum to apex of elytra (head is excluded to avoid measurement errors caused by its variable position). RL (rostrum length): measured in frontal view from anterior margin of eyes to anterior margin of rostrum or apex. RW (rostrum width): measured in frontal view immediately anterior to eyes or base of rostrum. PL (pronotum length): measured in dorsal view along midline. PWa (pronotum width at anterior margin). PWp (pronotum width at posterior margin). EL (Elytra length): measured along suture. EW (Elytra width): maximum width.

### 2.3. Distribution Map

Locality data were obtained from the specimen labels. The occurrence map was created in QGIS 3.22 software, based on the shapefile of the biogeographic regions of the Neotropics according to Morrone [17].

### 2.4. Morphology-Based Phylogenetic Analysis

A total of 60 adult morphological characters were selected and described with their coded states in Table 1. They were scored for 16 terminal taxa (Table S1). The internal group includes 11 species representing eight genera of Eudiagogini (*Aracanthus*, *Chileudius*, *Colecerus*, *Eudiagogus*, *Eudius*, *Eurysaces*, *Pororhynchus*, and *Promecops*), and the external group includes species representing closely related “leptopiine” tribes (Entimini, Strangaliodini, and Cylydrorhinini), and one species from the tribe Naupactini, which was used to root the cladogram. The specimens of the ingroup examined are deposited in the institutions mentioned above for the taxonomic study of *Eudius* and those of the outgroup taxa are in the MLP (Museo de La Plata, La Plata, Argentina). The data matrix of 16 terminal taxa and 60 morphological characters (Table S1) was analyzed under maximum parsimony using an exact search in TNT v 1.5 [18], considering all characters as disordered and under different weighting scenarios. Clade stability was evaluated by 1000 parsimony Jackknife replicates in TNT. The program WinClada [19] was used to optimize the apomorphies onto the selected cladogram (under “unambiguous”, “fast” and “slow” optimization options) and to prepare the MP tree figures.

### 2.5. Molecular Phylogenetic Analysis

The taxon sampling for the phylogenetic analysis based on molecular evidence was designed to be as compatible as possible with the taxon sampling of the morphology-based analysis (Table S2). Sequences were available for representative species of the same genera used as outgroups, and for some, though not all, of the ingroup taxa (*Eudius* and *Eurysaces* could not be included). When the same species coded for structural characters could not be sampled, it was replaced by one or more co-generic species for which sequences were available. A total of 18 terminal taxa were included, of which 10 correspond to ingroup taxa (in seven genera currently classified in tribe Eudiagogini), and eight are outgroup taxa (with *Naupactus* used to root the tree). The molecular data matrix in nexus format (Table S3) contains mitochondrial COI sequences (the barcode region COI-5P) for all the terminals and two nuclear ribosomal markers (18S and 28S-D2, D3) for a subset of these (outgroups and three genera of Eudiagogini). The approach of combining DNA barcodes with other markers sampled for a subset of representative taxa has proved helpful to assign taxa to genus or other higher-level groups [20]. The sequences used in this study (Table S2) are deposited in public databases: NCBI GenBank and/or BOLD. All were already available, except the 18S sequences (GenBank accession numbers PX570731 and PX570732) of the outgroup *Cylydrorhinus chilensis*, updated to complete the entire fragment, and of the ingroup terminal *Promecops claviger*, newly obtained for this study (same protocols as in Marvaldi et al. [9]). The sequences of ribosomal markers were aligned according to secondary structure of the gene [21], using as template the annotated alignment provided by Marvaldi et al. [9], as this approach objectively allows to recognize homologous positions while detecting regions of ambiguous alignment to be excluded from the analyses. A concatenated molecular data matrix (Table S3) was constructed for 18 terminal taxa, including character sets of 18S (positions 1–1922) for 10 taxa (7 outgroup and 3 ingroup terminals), 28S-regions D2 and D3 (positions 1923–2615) for 8 taxa (7 outgroup and 1 ingroup taxa) and COI barcode region (positions 2616–3273) for all the terminals (8 outgroup and 10 ingroup taxa).

Maximum parsimony (MP) analysis of the molecular data was performed in TNT [18]. For parsimony tree reconstruction, the third codon positions of COI were excluded and the gaps were treated as a fifth character state (additional analysis treating gaps as missing was also performed for comparison). The data matrix in nexus format was analyzed in TNT by means of a “traditional search”, with 1000 replicates, TBR (tree bisection reconnection) branch swapping, saving 100 trees per replicate, and collapsing trees after the search.

Maximum likelihood (ML) tree reconstruction analysis was performed with IQ-Tree [22], using IQ-Tree web server (iqtree.cibiv.univie.ac.at), with default parameters, using a partitioned scheme created with five partitions, two corresponding to each nuclear ribosomal marker and three to de codon positions of COI. The best substitution model for each partition was estimated using the ModelFinder algorithm [23], and the selected models according to BIC were K2P + I for 18S and 28S, TIM3e + G4 for COI-pos1, TPM3 + F + G4 for COI-pos2, and HKY + F + G4 for COI-pos3. Node support was evaluated with Ultrafast Bootstrap [24] and the SH-aLRT branch test [25]. An additional analysis was also performed on a data matrix without the third positions of COI (four partitions), for comparison.

## 3. Results and Discussion

### 3.1. Taxonomy

*Eudius* Schoenherr

*Eudius* Schoenherr, 1834: 162 [26], 1840: 441 (classification, diagnosis) [27]; Castelnau, 1840: 312 (diagnosis) [28]; Blanchard, 1851: 323 (classification, description) [29]; Lacordaire, 1863: 385, 388 (key, description) [5]; Schenkling & Marshall, 1931: 3 (catalog) [4]; Voss, 1934: 71, 74 (key, classification) [30]; Blackwelder, 1947: 806 (catalog) [31]; Wibmer & O’Brien, 1986: 81 (catalog) [1]; Alonso-Zarazaga & Lyal, 1999: 157 (catalog) [3]; Morrone, 1999: 119 (catalog) [32].

Type species. *Eudius quadrisignatus* Gyllenhal 1834, by original designation.

Diagnosis. *Eudius* is easily distinguished from other genera of Eudiagogini by the striped patterns on pronotum and elytra, connate tarsal claws, and vagina and bursa copulatrix with distinct sclerites.

**Figure 1:**
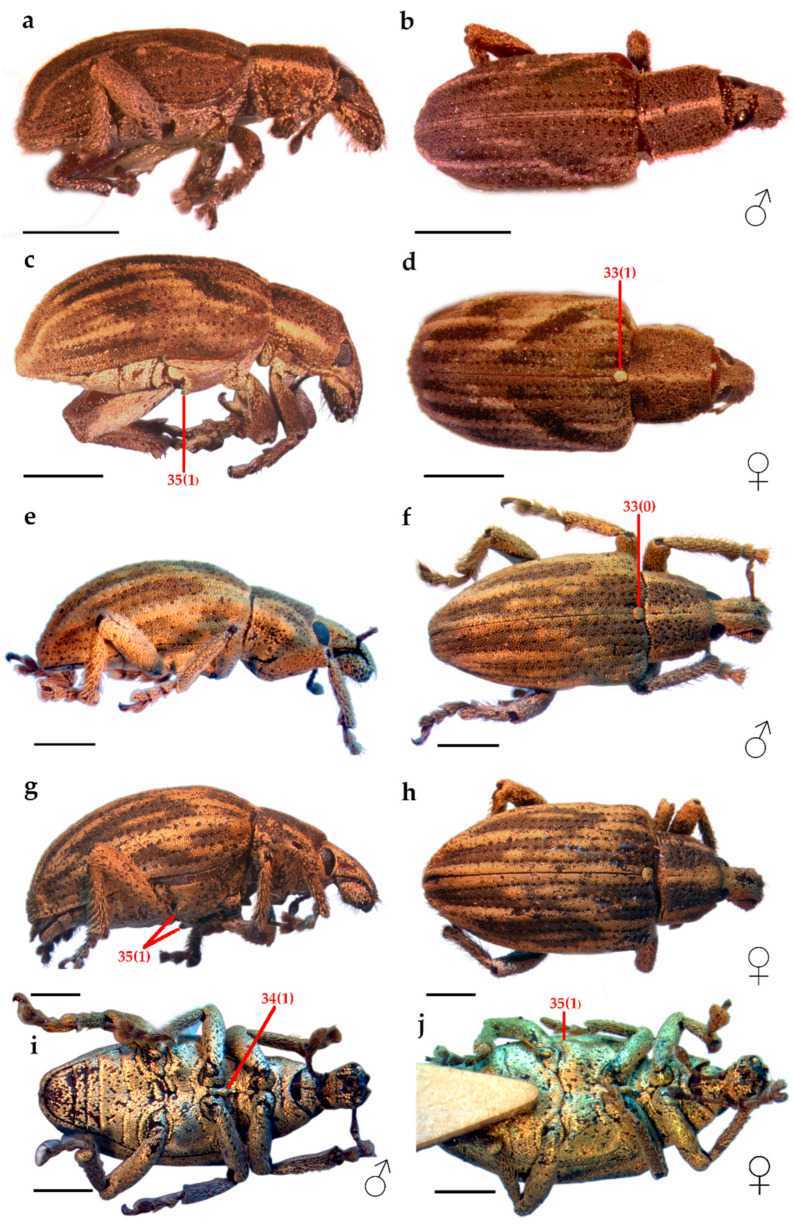
Habitus. *Eudius quadrisignatus*, male: (**a**) lateral view; (**b**) dorsal view, female: (**c**) lateral view; (**d**) dorsal view. *Eudius jocosus*, male: (**e**) lateral view; (**f**) dorsal view, female: (**g**) lateral view; (**h**) dorsal view; (**i)** ventral view male; (**j**) ventral view female. Scale bars = 1 mm.

Description.
Habitus (Figure 1). Medium-sized (3.5–6.0 mm). Body stout, broadly oval, maximum width about half TL, convex in lateral view. Integument reddish brown, covered with whitish to brownish or black scales, some iridescent, forming band patterns on pronotum and elytra; scales tessellate to imbricate, appressed, subcircular on dorsum and elongated on venter.

**Figure 2:**
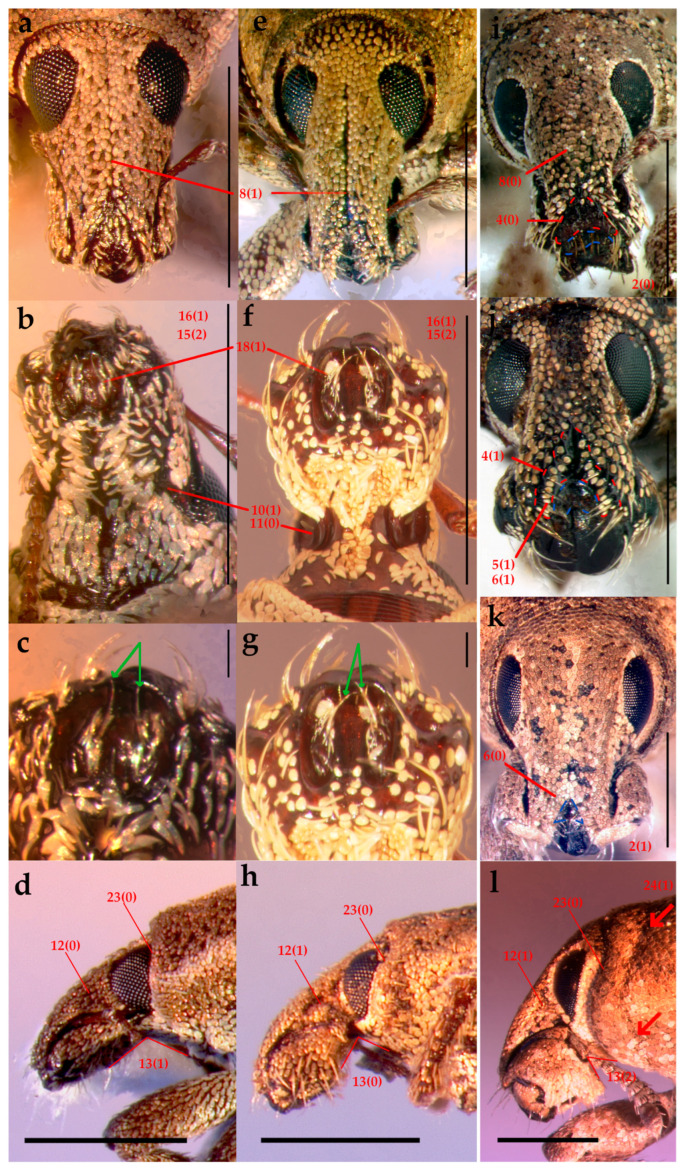
Head, rostrum, and mouthpart characters. *Eudius quadrisignatus*: (**a**) frontal view; (**b**) ventral view; (**c**) detail of maxilla showing setae diverging apically; (**d**) lateral view. *Eudius jocosus*: (**e**) frontal view; (**f**) ventral view; (**g**) detail of maxilla showing setae converging apically; (**h**) lateral view. *Chileudius varians*: (**i**) frontal view. *Eurysaces grammicus*: (**j**) frontal view. *Pororhynchus labeonis*: (**k**) frontal view; (**l**) lateral view. Scale bars = 1 mm, except (**c**,**g**) = 0.1 mm.

Head (Figure 2a,h). Rostrum stout, short, downcurved; linear median groove. Epistome subtriangular and sunken, mostly glabrous, with a posterior V-shaped area of sparse scales (different from rostral ones), distal setae elongated, decumbent, directed antero-mesad. Antennae: scape straight, distally inflated; club elliptical. Mouthparts: mandibles with whitish scales, and rigid, translucent, decumbent setae; maxillae partially exposed at sides of prementum (imperfectly adelognathous condition), bearing small, elongated scales and each with one elongate, suberect seta; prementum glabrous.

**Figure 3:**
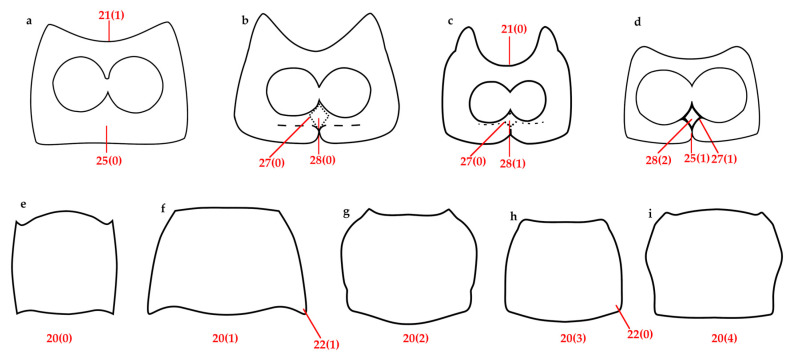
Diagram prosternum, junction of hypomeral lobes and sternellum: (**a**) *Naupactus xanthographus*; (**b**) *Vossius nebulosus*; (**c**) *Eudius jocosus*; (**d**) *Cylydrorhinus chilensis*. Diagram pronotum, shapes: (**e**) *Chileudius varians*; (**f**) *Pororhynchus albolateralis*; (**g**) *Eudiagogus episcopalis*; (**h**) *Eudius quadrisignatus*; (**i**) *Aracanthus mourei*.

**Figure 4:**
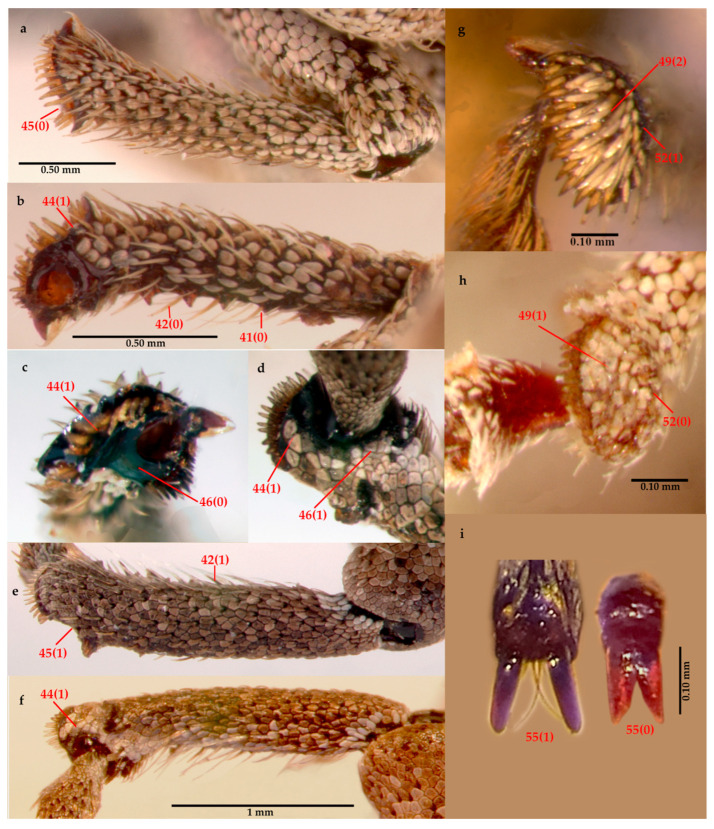
Leg characters. *Eurysaces grammicus*: (**a**) ectal surface; (**b**) ental surface; (**c**) apical surface. *Pororhynchus albolateralis*: (**d**) apical surface; (**e**) ectal surface; (**f**) ental surface. *Eudius jocosus*: (**g**) corbel setose and squamose. *Vossius nebulosus*: (**h**) corbel squamose, tessellate; (**i**) tarsal claws, *Pororhynchus labeonis* (left) and *Eudius quadrisignatus* (right).

Thorax (Figure 3c,h and Figure 4g,i [right]). Prothorax with flat flanks and ocular lobes. Pronotum with three light bands alternating with two (usually broader) dark bands; PL/PWa: 1.2, PWp/EW: 0.6, posterior margin bisinuate. Scutellar shield subpentagonal. Elytra elongate, about 3× longer than pronotum, EW/EL: 0.6, with a V-shaped color pattern, from humeri to about midlength, and with longitudinal discontinuous stripes on remaining areas; interstriae with white, sparse, recumbent, setiform scales; striae distinct, punctures bearing a minute elongated scale; humeri broadly rounded. Hindwings well developed. Mesoventrite with intermesocoxal process acute and anteriorly raised. Metaventrite swelling in front of metacoxae, acute and strongly produced; metacoxal distance approximately equal to length of first abdominal ventrite at middle. Legs: femora straight, with whitish setiform scales; metafemora medially inflated; tibiae slightly expanded distally, with setae increasing along inner margin, distal comb with short setae, corbel setose and scaly, tarsi with small (less than 0.25× the length of tarsomere 5) claws, connate at base.

**Figure 5:**
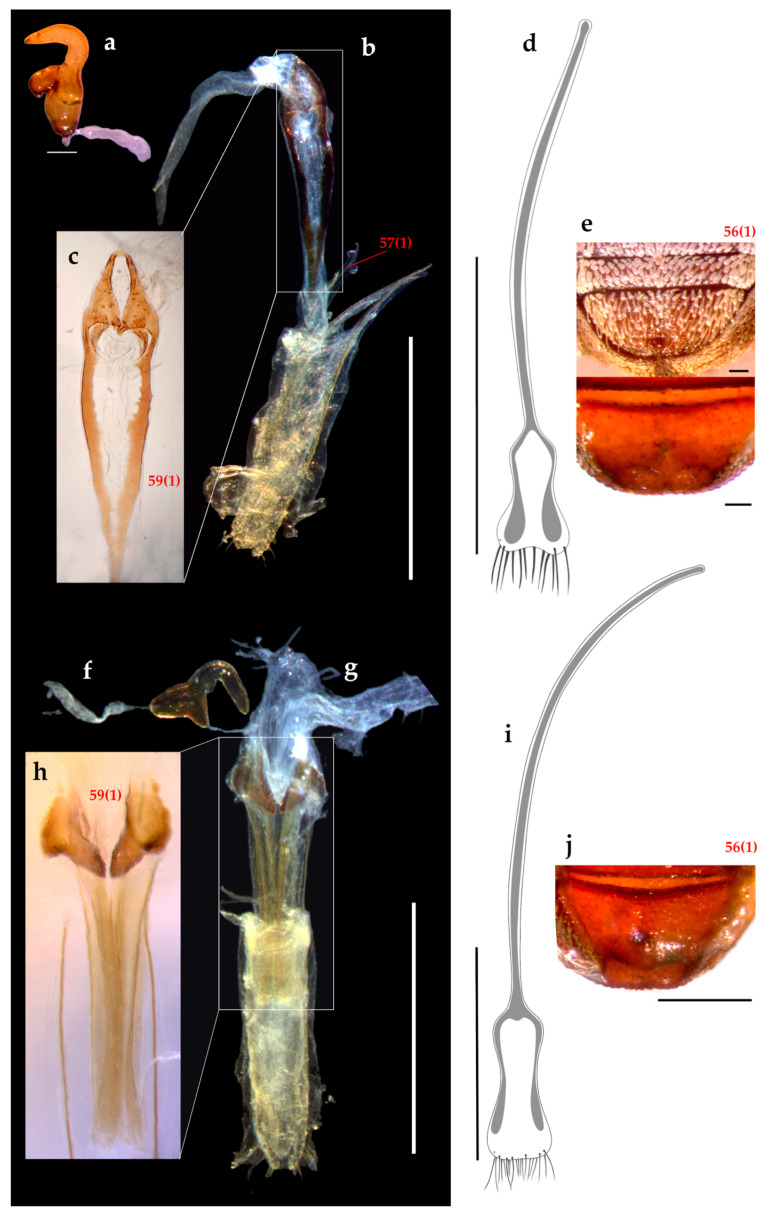
Female terminalia and ventrite 5. *Eudius quadrisignatus*: (**a**) spermatheca, scale bar = 0.1 mm; (**b**) ovipositor, scale bar = 1 mm; (**c**) detail of vagina and bursa copulatrix with sclerites; (**d**) sternite VIII, scale bar = 1 mm; (**e**) ventrite 5 showing a puncture in external view and a sclerite inside after clearing, scale bar = 0.1 mm. *Eudius jocosus*: (**f**) spermatheca; (**g**) ovipositor, scale bar = 1 mm; (**h**) detail of vagina and bursa copulatrix with sclerites; (**i**) sternite VIII, scale bar = 1 mm; (**j**) ventrite 5 showing a puncture and a sclerite inside after clearing, scale bar = 0.5 mm.

Abdomen (Figure 5e,j). Ventrites 1 and 2 subequal in length. Ventrite 2 longer than 3 + 4, these subequal. Ventrite 5 in females with a medial puncture near distal margin (better visible when cleared) and with an internal sclerite attached along the apical margin as in Figure 2e,j.

Female terminalia (Figure 5). Sternite VIII as long as ventrites 1–5, plate with wide lateral sclerotized arms almost reaching apex of plate, apical margin with row of long setae. Ovipositor with subparallel ventral baculi, vagina and bursa copulatrix with sclerites, styli absent.Spermatheca: ramus globose, spermathecal duct membranose.

**Figure 6:**
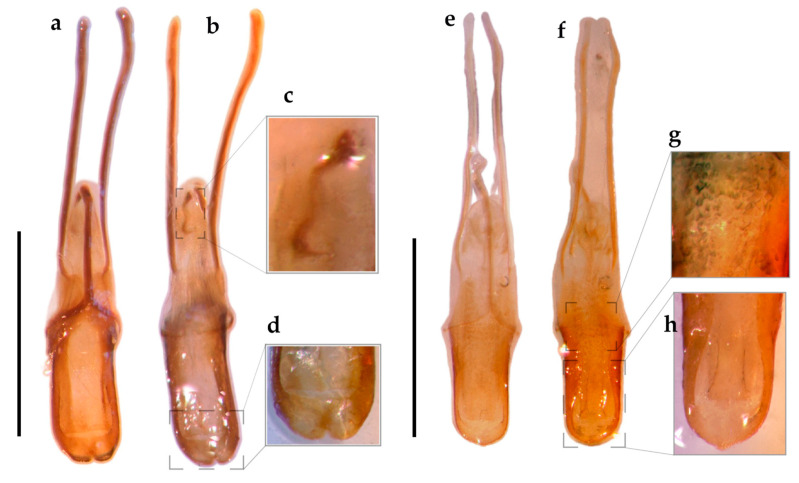
Male terminalia. *Eudius quadrisignatus*, aedeagus: (**a**) ventral view; (**b**) dorsal view; (**c**) detail of endophallus with sclerite; (**d**) detail of bilobate apical margin. *Eudius jocosus*, aedeagus: (**e**) ventral view; (**f**) dorsal view; (**g**) detail of endophallus armed with papillae; (**h**) detail of apical margin rounded with slightly pointed apex. Scale bars = 1mm.

Male genitalia (Figure 6). Penis as long as, or longer than, ventrites 1–5.

**Figure 7:**
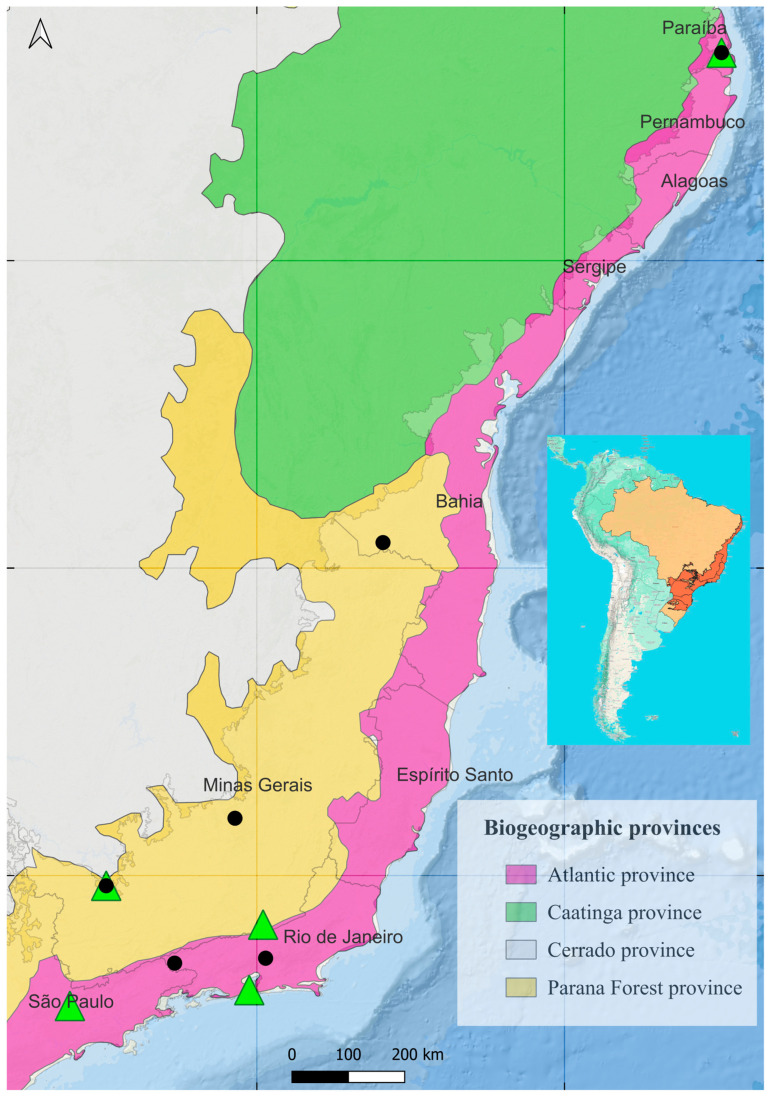
Map showing the distribution of *Eudius* species across biogeographic provinces in eastern Brazil. *Eudius quadrisignatus* (green triangle), *Eudius jocosus* (black circle).

Distribution (Figure 7). Brazil (Bahía, Espírito Santo, Minas Gerais, Paraíba, Rio de Janeiro, and São Paulo).

Biology. Unknown.

Included taxa. The genus only has two species: *Eudius quadrisignatus* and *E. jocosus*.

**Figure 8:**
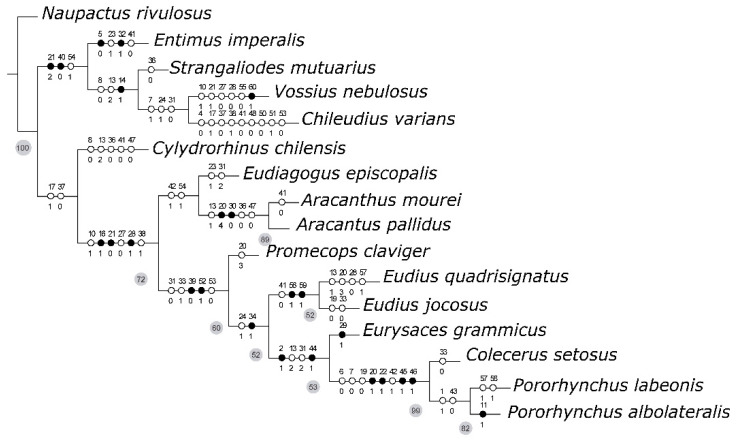
Most parsimonious tree of Eudiagogini, obtained after parsimony analysis of the morphological data (Table 1 and Table S1). Unambiguous character changes are indicated at branches, with numbers above and below corresponding to character and state, respectively; unique changes in black and homoplastic changes in white. Jackknife values over 50% are shown in grey circles.

Remarks. According to results of the cladistic analysis performed in this study (see Section 3.3.1 and Figure 8, Figures S1 and S2), the genus *Eudius* is a monophyletic group, supported by two exclusive synapomorphies: female ventrite 5 with a puncture located medially, internally forming a bilobate sclerite (Figure 5e,j), presence of sclerites in vagina and bursa of the female (Figure 5c,h), and also by some non-exclusive synapomorphies, like the absence of distinct crenulation with stout setae in internal margin of protibiae, maxillae partially covered with setae and scales, metatibial corbel with setose and scaly vestiture, and by tarsal claws connate at base. Within the clade Eudiagogini, *Eudius* is closely related (sister group) to a clade including *Eurysaces*, *Coelecerus* (probably also *Eucoleocerus*) and *Pororhynchus*.

*Eudius quadrisignatus* Gyllenhal 1834: 163

(Figure 1a–d, Figure 2a–d, Figure 3h, Figure 4i [right], Figure 5a–e and Figure 6a–d)

*Eudius quadrisignatus* Gyllenhal 1834 in Schoenherr, 1834: 163 [26]; Schoenherr, 1840: 441 (classification, diagnosis) [27]; Castelnau, 1840: 312 (diagnosis) [28]; Schenkling & Marshall, 1931: 3 (catalog) [4]; Blackwelder, 1947: 806 (catalog) [31]; Wibmer & O’Brien, 1986: 81 (catalog) [1]; Alonso-Zarazaga & Lyal, 1999: 157 (catalog) [3]; Morrone, 1999: 119 (catalog) [32].

Type material examined. Lectotype, male, herein designated, from Brazil, in shared pin (upper position), labeled as follows: “Eudius 4-signatus, Brasilia, paratypes”, handwritten (NHRS, col. Chevrolat). Paralectotypes: female, same pin as lectotype, lower position (NHRS, col. Chevrolat); one male and one female, in the same pin, labeled as follows: “Eud:4-signatus Campus Gerais, Brasilia. Chevrol. Typus. Allotypus”, handwritten (NHRS, col. Schoenherr).
  Additional material examined. **Brazil. Espírito Santo:** Schmidt coll., (1♀, NHMUK). **Minas Gerais:** *Mar de Espanha* [S21.866944°, W43.01°], J. Bechyné coll., 27–28/II/1962, (1♂ MZSP 56828). **Paraíba:** *Santa Rita?* [S7.113889°, W34.977778°], Sahlberg coll., (1, MTD). **Rio de Janeiro:** *Tijuca* [S22.9255°, W43.2521°], (1, HMMUK); Fry coll., (6, NHMUK). **São Paulo:** *Nazaré Paulista*, Faz. Araucaria, Malayse [S23.180833°, W46.395°], B.H. Dietz coll., 23/IX/2004, (1 MZSP 60460), (1 MZSP 60459). No locality data: (1, NHMUK).

Diagnosis. *Eudius quadrisignatus* differs from *E. jocosus* by the color pattern on pronotum and elytra; prothorax subcylindrical; rostrum with a superficial median groove, appearing as a faint linear impression; gular angle weak (>120°); antennal scape reaching anterior margin of eye; and ovipositor with ventral and dorsal baculi present.

Redescription.

Dimensions (mm): TL 3.4–4.5; RL 0.5–0.6, RW 0.5–0.6; PL 1.0–1.2, PWa 0.7–1.0, PWp 1.0–1.3; EL 2.7–3.6, EW 1.5–2.2.

Vestiture. Body covered with imbricate scales, light brown to black on dorsum, and light brown on venter and legs (Figure 1a–d).

Head (Figure 2a–d). **Rostrum** length subequal to RW, basal width equals to apical width of rostrum, gular angle weak (>120°), median groove shallow, gular suture raised. **Scrobes** apically spaced about half the width of rostral base. **Epistome** posteriorly ridged in ogival shape, with elongated setae at apex. **Ventral rostrum** with whitish, elongated scales, setiform scales and thin, short setae, all transverse from gular suture to sides. **Eyes** flat, subcircular, with posterior edge straight, subdorsal in position; interocular distance less than half the basal rostral width; anteocular depression faint. **Antennae**: **scape** reaching anterior eye margin; **funicle** segments 1–3 decreasing in length, 3–4 subequal, last three similar in length and progressively wider. **Mouthparts**: **mandibles** with dense, transversally oriented, elongated scales and thin, short setae; **maxillae** each with one translucent seta, setae diverge apically (Figure 2c).

Thorax (Figure 3h and Figure 4i [right]). **Prothorax** subcylindrical, weak postocular lobes, ventrally separated by a shallow emargination. **Pronotum** with two dark wide bands and sometimes paired black basal marks medially; subquadrate, PWa 0.8× narrower than PWp, length subequal to PWp, anterior margin straight. **Scutellar shield** flat, slightly longer than wide, with whitish, oval scales. **Elytra** with oblique bands from stria 6 (basally) to 3 (before midlength), jointly forming black V-shaped figure, cream-outlined; interstriae 5, 8 cream; interstriae 2, 4, 6, 7 black, variable in length; interstriae 5 and 8 in the posterior half more elevated; elytra in lateral view with dorsal outline more abruptly curved posteriorly. **Legs:** tibiae with translucid, short, thin, sparse setae, plus few spinelike setae and elongated scales; mucro present on meso- and metatibiae, indistinct on protibiae, corbel elliptic.

Abdomen. Ventrite 5 in males with a faint puncture situated at middle near distal margin, evident in KOH-cleared specimens.

Female terminalia (Figure 5a–e). **Sternite VIII:** apical margin bilobated; apodeme 3× longer than plate. **Ovipositor** slightly shorter (0.7×) than ventrites 1–5; ventral and dorsal baculi present, dorsal slightly shorter than ventral ones. Pair of conspicuous hook-shaped sclerites with spinose inner margin, between vagina and bursa copulatrix. **Spermatheca:** collum subcylindrical; ramus oriented 45° with respect to collum. Other characters as in generic description.

Male genitalia (Figure 6a–d). **Penis** 1.4× longer than ventrites 1–5; penis with apical margin bilobated; temones 1.3× longer than penis body; endophallus with sclerite (Figure 6b,c).

Distribution (Figure 7). Brazil (Espírito Santo, Minas Gerais, Paraíba, Rio de Janeiro, and São Paulo).

Remarks. According to results of the cladistic analysis (see Section 3.3.1 and Figure 8, Figures S1 and S2), the species *Eudius quadrisignatus* has the following autapomorphies: gular angle weak, >120° (13.1), pronotal shape subquadrangular (20.3), prosternal process “*Vossius*” like (28.0), ovipositor with dorsal baculi present (57.1), rostral median groove like a linear superficial impression (9.0), and (under fast optimization) indistinct rostral anteocular impression (12.0).

*Eudius jocosus* Fahraeus 1840

(Figure 1e–j, Figure 2e–h, Figure 3c, Figure 4g, Figure 5f–j, and Figure 6e–h)

*Eudius jocosus* Fahraeus 1840: 441.

*Eudius jocosus* Fahraeus 1840: 441 [27]; Schenkling & Marshall, 1931: 3 (catalog) [4]; Blackwelder, 1947: 806 (catalog) [31]; Wibmer & O’Brien, 1986: 81 (catalog) [1]; Morrone, 1999: 119 (catalog) [32].

Type material examined. Lectotype, herein designated, from Brazil, Minas Gerais, Campus Gerais, labeled as follows: “Eud: jocosus Chevrolat -unreadable word- Campos Gerais Chevrolat. Typus”, handwritten (NHRS, col. Schoenherr). In Chevrolat’s collection (at NHRS) we found three specimens labeled as types, which are probably not part of the type series. According with the original description, Campos Gerais was indicated in the labels, although this information is lacking for these specimens.

Additional material examined. **Brazil. Bahía:** *Encruzilhada*, 960 m [S15.5308°, W40.9089°], M. Alvarenga coll., XI-1972, (5♂, CWOB). **Espírito Santo:** (1♂, MTD), (1♀, MTD), (1♀, MNHM), (1, MFN), (1, MFN), Dr. Standnge coll., (1, MFN), (3, NHMUK). **Minas Gerais:** *Caraça* [S20.127918°, W43.502316°], Gounelle coll., 1/II/1885, (1♂, MNHM). **Paraíba:** *Santa Rita?* [S7.113889°, W34.977778°], Sahlberg coll. [S. Ripa Sahlberg], (1♂, MTD). **Rio de Janeiro:** *Teresópolis* [S22.411944°, W42.96583°], B. Pohl coll., XII/1955, (1♂, MZSP 57012), (1♂, MZSP 57013), (1♂, MZSP 57011), (1♀, MZSP 57022); *Itatiaia* [S22.491389°, W44.559167°], Diringa coll., II/1969, (1♀, MZSP 60485); De Castelnau coll., (1♀, MNHM), Fry coll., (7, NHMUK), (1, NHMUK). **No locality data:** (2♀, 5♂, MTD); Sicard coll. (1♀, 1♂, MNHM, labeled: “Brésil. Museum Paris 1930 Coll. Sicard. Eudius jocosus”); Chevrolat coll. (1, MFN, labeled: “Eudius jocosus Chevrolat Brasil. QR code MFN URI http://coll.mfn-berlin.de/u/0a0d30”); (1, MFN, labeled: “Eudius jocosus Fhrs. Det. E. VoB. Hist. -Coll. (Coleoptera) Nr.44548 Eudius iocosus Schh. Brasil., Virmd. Zool. Mus. Berlín. QR code MFN URI http://coll.mfn-berlin.de/u/09f37b”); (10, NHMUK). **No country data.** (4♂, 2 ♀, MACN), (3, MACN_col. Burmeister, labeled “Nov. Erib.”)

Diagnosis. *Eudius jocosus* differs from *E. quadrisignatus* by a more robust body; color patterns on pronotum and elytra more lustrous; prothorax subconical; rostrum with broad and deep median groove; gular angle strong (90–120°); and ovipositor only with ventral baculi.

Redescription.

Dimensions (mm): TL 4.2–6.0; RL 0.7–1.0, RW 0.5–0.7; PL 1.0–1.6, PWa 0.9–1.2, PWp 1.3–2.1; EL 3.0–5.2, EW 1.9–3.5.

Vestiture. Body covered with tessellate scales, light brown with a golden bronze glaze (Figure 1e–j).
Head (Figure 2e–h). **RL** about 1.4× RW, width slightly increases apically, gular angle strong (90–120°), median groove slightly deeper towards base, gular suture distinct but not raised. **Scrobes** apically spaced almost as wide as RW. **Epistome** posteriorly demarcated by subcircular scales and elongated setae arranged in a V-shaped pattern. **Ventral rostrum** with subcircular scales, anterior to scrobes globose scales and postero-anteriorly directed, thick, rigid, long setae, on postmentum similar setae but medio-laterally oriented. **Eyes** slightly convex, drop-shaped (with acute part towards venter), sublateral in position; interocular distance greater than half the basal rostral width; anteocular depression distinct. **Antennae**: **scape** not reaching anterior eye margin; **funicle** segments 1–5 decreasing in length, 5–6 subequal, 7 as long as 3, last three segments progressively wider. **Mouthparts**: **mandibles** with sparse, subcircular scales and thick, elongated setae; **maxillae** each with one whitish seta, setae converge apically (Figure 2g).
Thorax (Figure 3c and Figure 4g). **Prothorax** subconical, strong postocular lobes, ventrally separated by a deep emargination. **Pronotum** with two dark brown wide bands and sometimes a narrower lateral band on each side; trapezoidal, PWa 0.6× narrower than PWp, length 0.8× PWp, anterior margin slightly curved. **Scutellar shield** elevated, slightly wider than long, with same subcircular scales than pronotum. **Elytra** with interrupted longitudinal darker stripes: typically on interstriae 2–4 basally, 5–7 medially and 2–3 apical, leaving a V-shaped pattern of light brown scales; interstriae 4 and 7 the posterior half more elevated; elytra in lateral view with dorsal outline uniformly curved. **Legs:** tibiae with cream, long, thick, dense setae, mucro present on all tibiae in males, indistinct on metatibia in females; corbels subcircular.
Female terminalia (Figure 5f–j). **Sternite VIII:** with apical margin truncate; apodeme 3.3× longer than plate. **Ovipositor** as long as ventrites 1–5; only ventral baculi present. Vagina with 4 rods and pair of conspicuous conical sclerites at transitional zone between bursa copulatrix and vagina. **Spermatheca:** collum subconical, ramus oriented transversely to collum.
Other characters as in generic description.
Male genitalia (Figure 6e–h). **Penis** as long as ventrites 1–5; penis with apical margin rounded and slightly pointed apex; temones 1.6× longer than penis body; endophallus armed with papillae.

Distribution (Figure 7). Brazil (Bahía, Espírito Santo, Minas Gerais, Paraíba and Rio de Janeiro).

Remarks. According to results of the cladistic analysis (see Section 3.3.1 and Figure 8, Figures S1 and S2), the species *Eudius jocosus* has the following autapomorphies: antennal scape not reaching anterior margin of eyes (19.0), color of scutellar vestiture similar to that of elytra (33.0), and (under slow optimization): distinct rostral anteocular impression (12.1).

### 3.2. Distribution

The occurrence map (Figure 7) suggests that the distribution of *Eudius* specimens is restricted to the Atlantic Forest biome. Records exist for both species in five Brazilian states: Bahia, Espírito Santo, Minas Gerais, Paraíba, Rio de Janeiro, and, for *E. quadrisignatus*, also São Paulo.

### 3.3. Phylogenetic Analyses

#### 3.3.1. Morphological Evidence

The search for the most parsimonious tree (MPT) under implied weights (K = 15) produced a single tree (Fit: 3.2; CI: 0.6; RI: 0.7), which topologically coincides with one of the three MPTs obtained under equal weights (L: 124) (Figure 8), which is the preferred phylogenetic hypothesis. The MP tree in Figure 8 shows unambiguous optimization of adult morphological characters, with unique changes (=exclusive synapomorphies) in black, and with homoplastic changes (=non-exclusive synapomorphies) in white. Figures S1 and S2 show character changes according to fast and slow optimization, respectively. The monophyly of tribe Eudiagogini, excluding the genus *Chileudius*, is firmly justified by: ventrally delimited antennal scrobes (10.1), cavernous sunken prementum (**16.1**), anterior margin of prosternum deeply emarginate or notched (**21.0**), poorly defined anterior and posterior margins of process formed by hypomeral lobes and sternellum (27.0), prosternal process of *Eudiagogus* type (**28.1**), presence of a scale at each puncture of elytral stria (38.1), additionally (under fast optimization) by: rostral length less than 1× its width (1.1), presence of a spine-like swelling anterior to each metacoxa on metaventrite (**35.1**), tibial apex with ascending comb well defined and oblique to apical comb (**40.1**), and also (under slow optimization) by precoxal and postcoxal areas of subequal lengths (29.2). This morphology-based phylogenetic analysis confirms the membership of *Eudius* in the tribe Eudiagogini and supports the monophyly of the genus, backed by the synapomorphies: presence in female ventrite 5 of a puncture located medially, internally forming a bilobate sclerite as in Figure 5e,j (**56.1**), presence of sclerites in vagina and bursa of the female, as in Figure 5c,h (**59.1**), absence of distinct crenulation with stout setae in internal margin of protibiae (41.0), also (under slow optimization) by maxillae partially covered with setae and scales (18.1), corbel of metatibia with setose and scaly vestiture (49.2) and tarsal claws connate at base (55.0). The sister group relationship of *Eudius* with a clade composed of the genera *Eurysaces*, *Pororhynchus*, and *Colecerus*, is suggested by the synapomorphies: presence of intercoxal process in mesoventrite (**34.1**) and prothoracic collar complete (24.1). Monophyly of *Eudius*’s sister group is supported by several synapomorphies, among them apically widened rostrum (**2.1**) and protibia with subapical or apical external surface modified into a flat lenticular surface, as in Figure 4d,f (**44.1**). The close relationship between the genera *Pororhynchus* and *Colecerus* is supported by several synapomorphies, among which they are worth to mention: subtrapezoidal shape of pronotum (**20.1**), projected posterolateral angles of pronotum (**22.1**), presence of subapical notch in protibia (**45.1**) and presence of scales on the denticular area of protibial apex (**46.1**).

#### 3.3.2. Molecular Evidence

**Figure 9:**
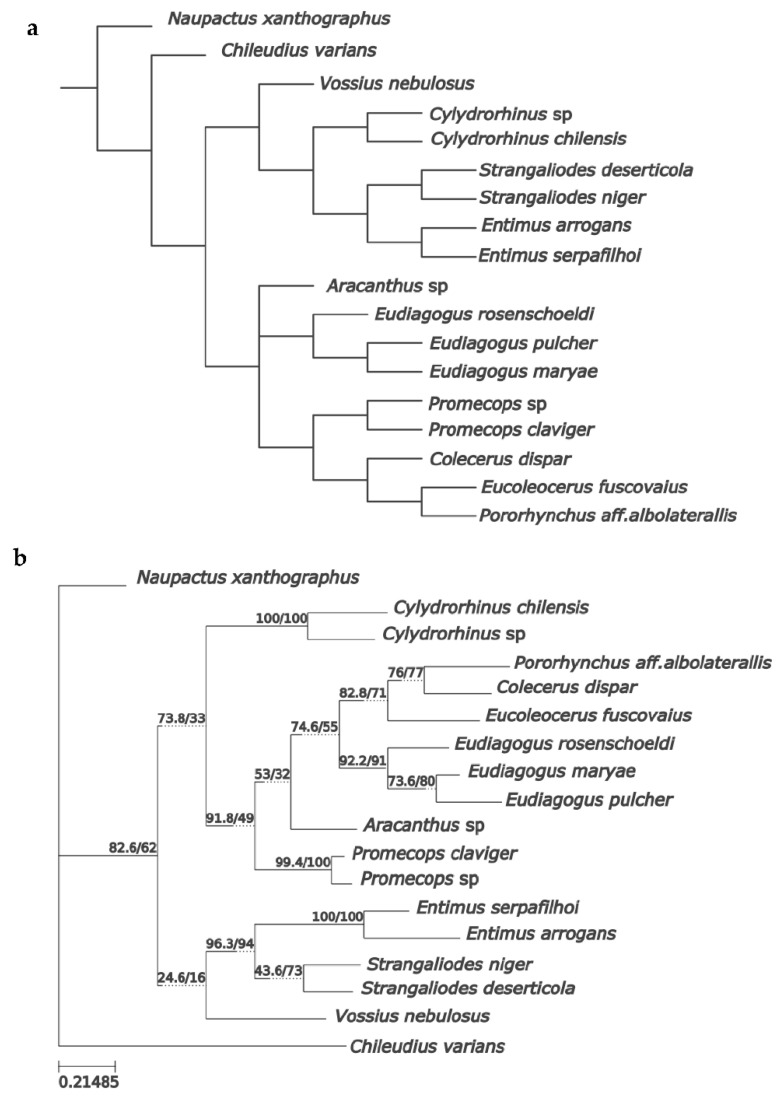
Phylogenetic trees of Eudiagogini, resulting from analyses of the molecular data (Tables S2 and S3). (a) Maximum parsimony tree. (b) Maximum likelihood tree with SH-aLRT/UFBoot support values on branches.

Results of phylogenetic analyses of the molecular data set using different optimality criteria (maximum parsimony and maximum likelihood) are shown in Figure 9a,b. Although results of MP and ML are not identical in some intergeneric relationships (albeit without significant support), they agree in recovering monophyly of the genera represented by more than one species, in outgroup and ingroup. More importantly, they are congruent in recovering the monophyly of the tribe Eudiagogini with exclusion of the genus *Chileudius*, which is fully consistent with the results based on morphology. Statistical support values were expected to be low considering that for about 50% of the taxa (most ingroup terminals), the COI barcode was the only marker available.

## 4. Concluding Remarks

In this contribution, and as part of a wider systematic project on Neotropical weevils of the tribe Eudiagogini, we performed a taxonomic revision of the genus *Eudius* and provided the first insights into the phylogeny of the tribe. On the basis of adult morphology and the available molecular evidence from the COI barcode and two nuclear ribosomal markers, we tested the monophyly of the tribe Eudiagogini, as well as the position of *Eudius* within it. A natural concept of the tribe is now available, excluding the genus *Chileudius*, which is here placed as *incertae sedis* in Entiminae, pending further analyses. Future research and collaborative studies with more complete taxon and character sampling will certainly help advance towards a natural system in Entiminae. We expect that integrating taxonomic and phylogenetic approaches to other genera will finally help to resolve many of the uncertainties posed by the “leptopiine” weevils.

## Figures and Tables

**Table 1:** List of morphological characters and states, used in the cladistics analysis of Eudiagogini.

ID	Description
1	Rostrum, length relative to width taken at the anterior margin of eyes: equal to or greater than one times the width (0) (Figure 1); less than one times the width (1)
2	Rostrum, maximum width at apex compared to width at base: equal to or narrower than the base (0) (Figure 2i); expanded apically (1) (Figure 2k)
3	Rostrum, epistome, maximum width relative to distance between scrobes at apex: ≤1/3 (0); >1/3 (1)
4	Rostrum, posterior region of epistome, vestiture: absent (0) (Figure 2i); present (1) (Figure 2j)
5	Rostrum, posterior region of epistome, vestiture type: setae (0); scales (1) (Figure 2j)
6	Rostrum, posterior region of epistome, size, density and orientation of scales compared to those of the rest of the rostrum: homogeneous (0) (Figure 2k); heterogeneous (1) (Figure 2j)
7	Rostrum, posterior region of epistome, demarcated posteriorly by a change in the surface of the naked integument: absent (0); present (1)
8	Rostrum, median groove: absent or indistinct (0) (Figure 2i); present (1) (Figure 2a,e)
9	Rostrum, median groove, shape and depth: like a linear impression, superficial (0); like a broad groove (wider than 1/3 the distance between eyes); deep (1)
10	Rostrum, scrobes ventrally delimited: no (0); yes (1) (Figure 2b)
11	Rostrum, scrobes vestiture ventrally: absent (0) (Figure 2b); present (1)
12	Rostrum, lateral view, anteocular depression: indistinct (0) (Figure 2d); distinct (1) (Figure 2h,l)
13	Head, lateral view, gular angle: marked (90–120°) (0) (Figure 2h); weak (>120°) (1) (Figure 2d); acute (<90°) (2) (Figure 2l)
14	Head, vertex (posterior dorsal area of the head, anterior to the eyes), groove as a line: absent (0); present (1)
15	Mouthparts, condition according to the extent to which prementum covers the maxillae: adelognathous, maxillae completely concealed (0); imperfectly adelognathous, maxillae partially concealed (1); phanerognathous, maxillae exposed continuously at sides of prementum (2) (Figure 2b,f)
16	Mouthparts, labium, prementum, cavernous (sunken) appearance: absent (0); present (1) (Figure 2b,f)
17	Mouthparts, maxilla, vestiture of stipes and/or palpifer: absent (0); present (1)
18	Mouthparts, maxilla, vestiture of stipes and/or palpifer: setae (0); setae and scales (1)
19	Antennae, scape, relative length: not reaching the anterior margin of eyes (0); reaching the anterior margin of eyes (1)
20	Pronotum, shape: sub-circular (curved sides, maximum width near the middle) (0) (Figure 3e); sub-trapezoidal (1) (Figure 3f); sub-hexagonal (divergent sides towards the middle or first third and then sub-parallel) (2) (Figure 3g); sub-quadrangular (3) (Figure 3h); cup-shaped (4) (Figure 3i)
21	Prosternum, anterior margin, shape: deeply emarginated or notched (0) (Figure 3c); linear, almost straight (1) (Figure 3a); slightly emarginate (2)
22	Pronotum, postero-lateral angles: not projected (0) (Figure 3h); projected (1) (Figure 3f)
23	Prothorax, postocular lobes, shape: curved (0) (Figure 2d,l); angular (1)
24	Prothorax, collar, delimitation: incomplete, only laterally distinct (0); complete, dorsally and laterally bounded (1) (Figure 2l)
25	Prothorax, prosternum, suture or separation where hypomeral projections meet: absent (0) (Figure 3a); present (1) (Figure 3d)
26	Prothorax, prosternum, sternellum, elevation relative to surface of prosternum: flat (0); raised (1)
27	Prothorax, prosternum, hypomeral lobes and sternellum (as a process), outline of the posterior and anterior margins: poorly defined (0) (Figure 3b,c); well defined (1) (Figure 3d)
28	Prothorax, prosternum, hypomeral lobes and sternellum (as a process): *Vossius* type (0) (Figure 3b); *Eudiagogus* type (1) (Figure 3c); *Cylydrorhinus* type (2) (Figure 3d)
29	Prothorax, prosternum, ratio between lengths of its precoxal and postcoxal areas, measured by midline of coxae: precoxal area shorter than postcoxal (0); precoxal area longer than postcoxal (1); sub-equal (2)
30	Mesothorax, scutellum, visibility: indistinct or barely visible (0); clearly visible (1)
31	Mesothorax, scutellum, shape: sub-pentagonal, sub-square or round (0); sub-triangular (1); sub-rectangular, sub-trapezoidal (2)
32	Mesothorax, scutellum, vestiture: scaly (0); bristly (1)
33	Mesothorax, scutellum, color of vestiture relative to that of elytra: similar (0) (Figure 1f); different (1) (Figure 1d)
34	Mesothorax, mesoventrite, intercoxal process: absent (0): present (1) (Figure 1i)
35	Metathorax, metaventrite, spine-like swelling anterior to metacoxa: absent (0); present (1) (Figure 1c,g,j)
36	Elytra, humeri: rounded, not produced (0); clearly projected (1)
37	Elytra, interstria, ratio of its width relative to width of stria: at least 3 times wider than stria (0); equal to or less than width of stria (1)
38	Elytra, stria, a scale at each puncture: absent (0); present (1)
39	Legs, tibial apex, ascending comb: absent (0); present (1)
40	Legs, tibial apex, ascending comb: slightly differentiated from apical comb (0); well-defined, oblique to apical comb (1); well-defined, transverse to apical comb (2)
41	Legs, protibia, internal margin, crenulation with stout setae: absent or indistinct (0) (Figure 4b); present, evident (1)
42	Legs, protibia, color of stout setae in the internal margin: translucent or yellowish (0) (Figure 4b); black to dark brown (1) (Figure 4e)
43	Legs, protibia, distribution of stout setae in the internal margin: in the apical 2/3 (0); in the apical 3/4; in the apical 1/3 (2)
44	Legs, protibia, flat lenticular area apical or subapical on external side: absent (0); present (1) (Figure 4b–d,f)
45	Legs, protibia, subapical notch: absent (0) (Figure 4a); present (1) (Figure 4e)
46	Legs, protibia, apex, scales on the articular area: absent (0) (Figure 4c); present (1) (Figure 4d)
47	Legs, metatibia, apex, corbel: absent (0); present (1)
48	Legs, metatibia, corbel, vestiture: absent (0); present (1)
49	Legs, metatibia, corbel, vestiture type: setose (0); squamose (tessellate) (1) (Figure 4h); setose and squamose (2) (Figure 4g)
50	Legs, metatibia, corbel, width: narrow (0); wide (1)
51	Legs, metatibia, corbel, angle formed by the inner edge with respect to the outer edge: <45° (almost horizontal) (0); >45° (more vertical) (1)
52	Legs, metatibia, corbel, outer edge as a keel: absent (0) (Figure 4h); present (1) (Figure 4g)
53	Legs, metatibia, corbel, outer comb: absent (0); present (1)
54	Legs, metatibia, apical margin, shape: straight (0); angled or bent (1)
55	Legs, tarsal claws: connate at base (0) (Figure 4i); free (1) (Figure 4i)
56	Female abdomen, ventrite 5, median pit: absent (0); present (1) (Figure 5j)
57	Female genitalia, ovipositor, dorsal baculi: absent (0); present (1) (Figure 5b)
58	Female genitalia, ovipositor, styli: absent (0); present (1)
59	Female genitalia, bursa, sclerites: absent (0); present (1) (Figure 5c,h)
60	Male genitalia, endophallus, flagellum: absent (0); present (1)

## Data Availability

The original contributions presented in this study are included in the article’s main text or Supplementary Information. Further inquiries can be directed to the corresponding authors.

## Supplementary Materials

The following supporting information can be downloaded at https://www.mdpi.com/article/10.3390/insects16121278/s1, Figure S1: Most parsimonious tree of Eudiagogini, with morphological character changes under fast optimization. Figure S2: Most parsimonious tree of Eudiagogini, with morphological character changes under slow optimization. Table S1: Morphological data matrix of 16 terminal units and 60 characters. Table S2: Terminal taxa for phylogenetic analyses based on data from DNA sequences, with accession codes. Table S3: Molecular concatenated data matrix of 18S rDNA and 28S rDNA sequence alignments without the RAA (regions of ambiguous alignment) and COI-5P.
