# Supplementary material for: Taxonomic Revision of the South American Genus Eudius and First Insights into the Phylogeny of the Tribe Eudiagogini (Curculionidae: Entiminae)
Source: Insects. 2025 Dec 16;16(12):1278. doi: 10.3390/insects16121278 (PMC12733461; doi:10.3390/insects16121278)
Supplement: Supplementary file 1 [file insects-16-01278-s001.zip › TableS2_MOL_Taxa_sampled_Eudiagogini.pdf]

Tribe	Genus-species		Locality	18S	28S	COI-5P
**Naupactini**	*Naupactus xanthographus (Germar)*		Argentina, Mendoza	FJ867775	FJ867695	NC_018354
**Cylydrorhinini**	*Cylydrorhinus chilensis* (Blanchard)		Argentina, La Rioja, Dto. Vinchina	**PX570731**	MH746335	MH734202
	Cylydrorhinus sp.		Argentina, Jujuy, Dto. Susques	MH746287	MH746336	MH734203
**Entimini**	*Entimus serpafilhoi* Morrone et al.		Brazil, Rio de Janeiro, Coastal Restingas	MH746327	MH746377	MH734211
	Entimus_arrogans Pascoe		Costa Rica, Alajuela, Zapote Upala	-	-	SICOA662-18
**Strangaliodini* (Leptopiini)**	*Vossius nebulosus* (Gyllenhal)		Argentina, Misiones, on way to Salto Tabai, close to Jardin America	MH746323	MH746372	MH734210
	Strangaliodes niger (Blanchard)		Argentina, San Juan	MH746326	MH746376	MH734209
	Strangaliodes deserticola Kuschel		Chile, Atacama	MH746325	MH746374	MH734207
**Eudiagogini**	*Pororhynchus aff. albolateralis* Hustache		Argentina, San Juan, near Telteca	MH746288	MH746337	MH734212
	Eudiagogus rosenschoeldi Fahraeus		GenBank source (United States of America)	AF250081	-	HM433165
	Eudiagogus_maryae Warner		USA, Florida, Sarasota	-	-	BBCCA1399-12
	Eudiagogus_pulcher Fahraeus		USA, Texas, Brazos Bend State Park	-	-	BBCCA1396-12
	Promecops_claviger Hustache / *P.* sp. *MLP1011-11*		Argentina, Tucumán, EEAOC, Dept. Cruz Alta [18S] / Misiones, PP. Moconá [COI]	**PX570732**	-	MLP1011-11
	Promecops sp. *GMARP968-14*		Argentina, Misiones, Oberá	-	-	OM609769
	Colecerus_dispar (LeConte)		USA, Arizona, Cochise Stronghold	-	-	CNCCF019-12
	Eucoleocerus_fuscovarius Champion		Mexico, Chiapas, Aguacera	-	-	SICOA803-18
	Aracanthus sp.		USA, Mississippi, Sharkey	-	-	SICOA797-18
	Chileudius_varians (Blanchard)		Chile, Linares, Malcho	-	-	SICOA798-18
