# Supplementary figures and images for: Taxonomic Revision of the South American Genus Eudius and First Insights into the Phylogeny of the Tribe Eudiagogini (Curculionidae: Entiminae)
Source: Insects. 2025 Dec 16;16(12):1278. doi: 10.3390/insects16121278 (PMC12733461; doi:10.3390/insects16121278)

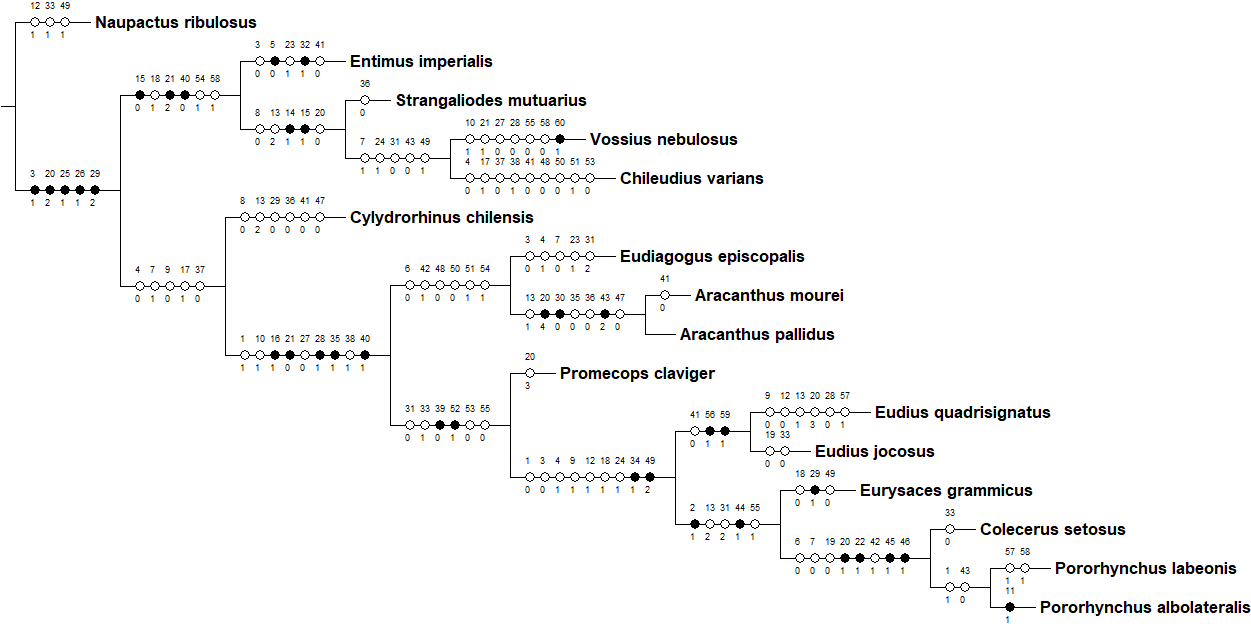

Supplement: Supplementary file 1 [file insects-16-01278-s001.zip › Figure S1 MPtree fast.tif]

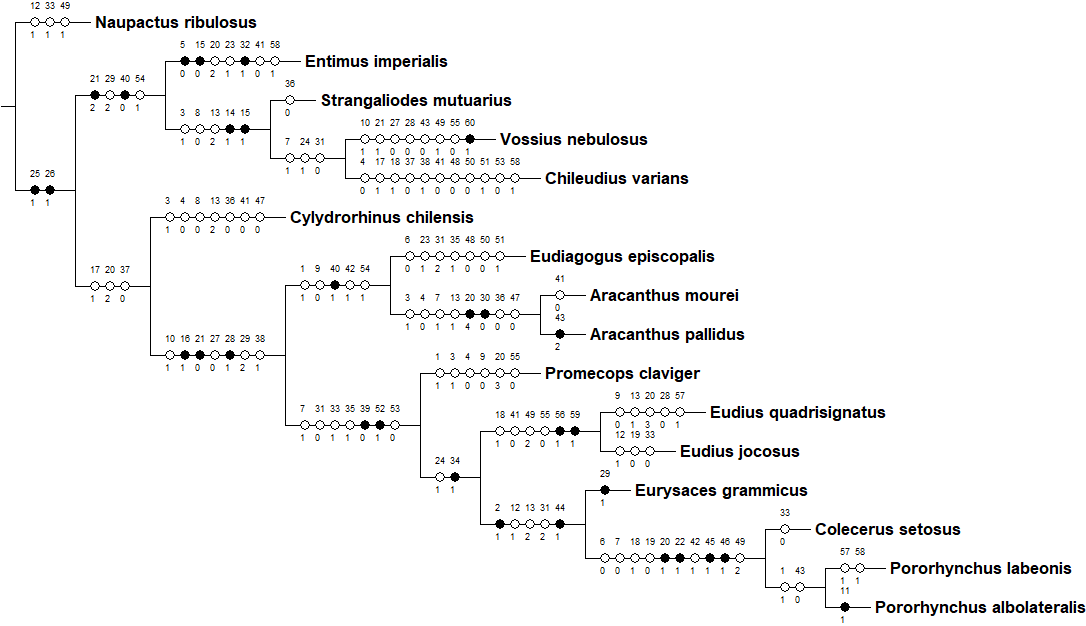

Supplement: Supplementary file 1 [file insects-16-01278-s001.zip › Figure S2 MPtree slow.tif]
